# Model-Based Sensitivity Analysis of the Temperature in Laser Powder Bed Fusion

**DOI:** 10.3390/ma17112565

**Published:** 2024-05-27

**Authors:** Zhihao Yang, Shiting Zhang, Xia Ji, Steven Y. Liang

**Affiliations:** 1 School of Mechanical Engineering, Donghua University, Shanghai 201620, China; 2211007@mail.dhu.edu.cn (Z.Y.); 2211026@mail.dhu.edu.cn (S.Z.); 2 Woodruff School of Mechanical Engineering, Georgia Institute of Technology, Atlanta, GA 30332, USA; steven.liang@me.gatech.edu

**Keywords:** laser powder bed fusion (LPBF), temperature history, analytical model, molten pool

## Abstract

To quantitatively evaluate the effect of the process parameters and the material properties on the temperature in laser powder bed fusion (LPBF), this paper proposed a sensitivity analysis of the temperature based on the validated prediction model. First, three different heat source modes—point heat source, Gaussian surface heat source, and Gaussian body heat source—were introduced. Then, a case study of Ti6Al4V is conducted to determine the suitable range of heat source density for the three different heat source models. Based on this, the effects of laser processing parameters and material thermophysical parameters on the temperature field and molten pool size are quantitatively discussed based on the Gaussian surface heat source. The results indicate that the Gaussian surface heat source and the Gaussian body heat source offer higher prediction accuracy for molten pool width compared to the point heat source under similar processing parameters. When the laser energy density is between 40 and 70 J/mm^3^, the prediction accuracy of the Gaussian surface heat source and the body heat source is similar, and the average prediction errors are 4.427% and 2.613%, respectively. When the laser energy density is between 70 and 90 J/mm^3^, the prediction accuracy of the Gaussian body heat source is superior to that of the Gaussian surface heat source. Among the influencing factors, laser power exerts the greatest influence on the temperature field and molten pool size, followed by scanning speed. In particular, laser power and scan speed contribute 38.9% and 23.5% to the width of the molten pool, 39.1% and 19.6% to the depth of the molten pool, and 38.9% and 21.5% to the maximum temperature, respectively.

## 1. Introduction

Laser powder bed fusion (LPBF) is a method of manufacturing that involves the use of lasers to fuse powdered materials together. It is commonly utilized because of its exceptional capacity for mechanical shaping in industry [1,2,3,4,5,6]. However, the quality of the built pieces is mostly affected by the thermal history that the material undergoes during the manufacturing process, while the temperature history can be influenced by many factors, such as process parameters, material properties, scan strategies, and so on [7,8,9]. Therefore, it is very important to quantitatively evaluate the influencing factors of the temperature for the quality prediction and control of the build parts.

Throughout the LPBF process, the laser melts metal powder particles, generating a large amount of heat that forms a molten pool. The LPBF encompasses intricate heat and mass transmission mechanisms, such as conduction, convection, radiation, and evaporation [10]. Many studies have been focused on the study of temperature during the LPBF process.

Zhao et al. [11] analyzed the thermal behavior of 7075 aluminum alloy by the FEM method and found that the size of the molten pool expanded as the laser power increased. They also studied the relationship between the process parameters and the microstructure evolution. Anand et al. [12] built an extensive model to forecast the size and properties of the melt pool. They conducted preliminary numerical simulations to acquire transient distributions of melt pool temperature and velocity in the SLM process, which can identify the transition from conduction mode to hole locking mode. Wang et al. [13] developed a novel computational model to forecast the melt pool characteristics, considering the material evaporation and a modified heat source in LPBF of Ti6Al4V, which is very fast, and it requires only 1/100th of the time taken by the fluid flow model.

From the above literature review, it is indicated that the temperature in LPBF is influenced by process parameters and material thermophysical properties, such as laser power, scan speed, specific heat, and so on. However, most studies relied on experimental and finite element methods, which is both time-consuming and costly. A method for the accurate and rapid prediction of the temperature during LPBF must be developed. Yang et al. [14] conducted a study where they examined the impact of various laser source geometries on the estimation of thermal history in a thermal model using an analytical approach. This significantly reduces computational time and cost. Liu et al. [15] examined the relationship between the anticipated shape of the melted area and the thresholds for inadequate melting and lock-hole transition defects, and proposed an analytical model which can quickly predict the melt pool cross-section geometry and determine the process window. Liu et al. [16] devised an analytical model to forecast porosity in selective laser melting (SLM), enabling calculation of the melt pool size. Korhan et al. [17] developed a mathematical model to determine the process parameters for manufacturing powder materials through selective laser melting using the eigenfunction expansion method.

From the discussion above, it is found that previous studies are mainly focused on the qualitative discussion of the relationship among the material properties, process parameters, and the melt pool [18], but there are few quantitative discussions, while quantitative discussion is of great value to the process planning of the build parts.

Based on the analytical modeling of the temperature in LPBF which was conducted by Wang et al. [19], this paper proposes a sensitivity analysis of the temperature in LPBF. First, the physical phenomena of LPBF are illustrated. And, then, three different heat sources—point heat source, Gaussian surface heat source, and Gaussian body heat source—are introduced in detail in order to determine the applicable range of the heat source density for different heat sources. After that, a sensitivity analysis of the temperature in LPBF is quantitively evaluated, including the impacts of the process parameters and the material properties.

## 2. Methods

### 2.1. Physical Phenomena of Laser Powder Bed Fusion

**Figure 1:**
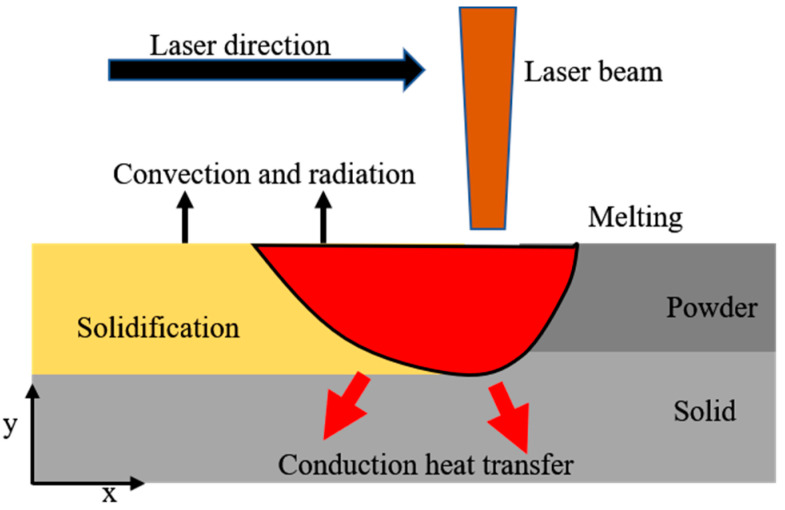
Physical phenomena in LPBF.

During the process of LPBF, the laser heat source acts on the metal powder, causing it to melt and create a molten pool [20]. The molten pool’s upper surface and the scanned region experience heat radiation and thermal convection. Simultaneously, the thermal energy emanating from the molten pool transfers to the nearby powder and the substrate by means of heat conduction [21,22]. This detailed exposition unveils the fundamental thermal characteristics and physical phenomena of the selective zone laser melt-forming process as shown in Figure 1.

### 2.2. Analytical Modeling of LPBF

#### 2.2.1. Heat Transfer Analysis

The heat exchange analysis is crucial to the temperature prediction in LPBF. To streamline and enhance the clarity of the model, some assumptions are made as following:

(1) The physical properties of the material are isotropic and remain constant, and are independent of the temperature; (2) the metal powder layer is considered as a regular geometry with uniform thickness, the porosity between the powders is not considered, and the powder layer is considered as a solid; (3) the heat flux resulting from convection and radiation on the top surface is negligible when compared to conduction in a semi-infinite solid, while the deposited layer and the substrate are considered as a whole.

In a homogeneous solid, the heat transfer is controlled by a linear partial differential equation during LPBF:

(1) $$\frac{\partial u\rho }{\partial t}+\frac{\partial \rho \Delta hV}{\partial x}=\nabla \cdot (k\nabla T)+\dot{q}$$

where u denotes internal energy, Δ*h* is enthalpy, ρ denotes density, k denotes thermal conductivity, $\dot{q}$ is a heat source, *T* denotes temperature, and *V* is heat source velocity [23]. The left side of the equation consists of two terms. The first term represents the change in internal energy, while the second term represents the convection component. The primary term on the right side of the equation denotes the thermal conductivity, while the second term designates the heat source component [24,25].

**Figure 2:**
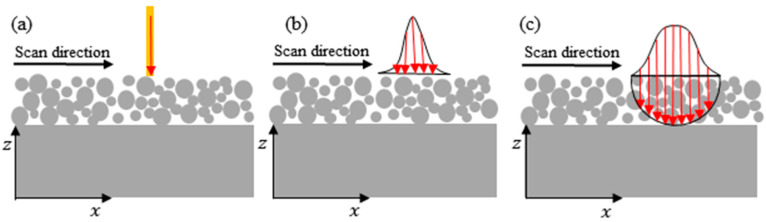
Three heat source models: (**a**) point heat source; (**b**) Gaussian surface heat source; (**c**) hemispherical Gaussian body heat source.

The three heat source models, point heat source model, the Gaussian surface heat source model, and the hemispherical Gaussian body heat source, are introduced in this paper, as shown in Figure 2.

The first step is to obtain the analytical solution of the temperature distribution caused by a point heat source in LPBF.

**Figure 3:**
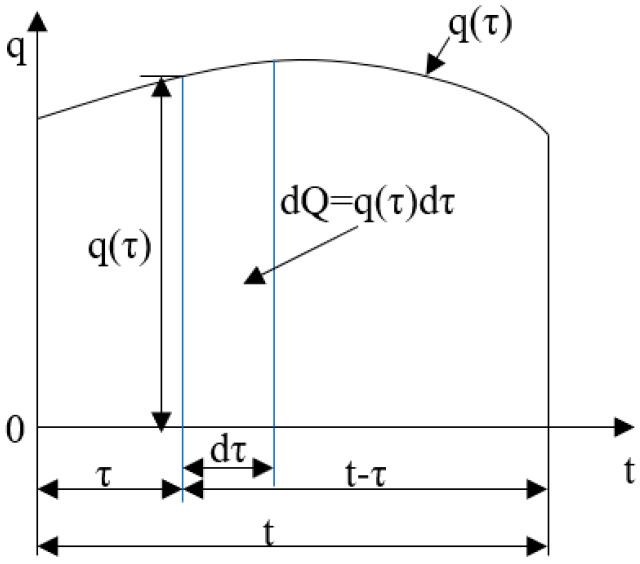
Schematic time axis of heat source action.

Consider a point source of heat traveling with a velocity *V* across the upper surface of a semi-infinite medium. The power of this source is *P*. Positioned at the origin within the dynamic Cartesian coordinate system and moving linearly along the *x*-axis, as depicted in Figure 3, the heat quantity can be expressed as dQ=Pdτ, where τ is a dimensionless time variable defined as $\tau =kt/R^{2}$; the thermal diffusivity *D* can be calculated as D=k/ρc [19,26].

(2) $$T(R,t)=\frac{Q}{4c\rho {\left(\pi Dt\right)}^{3/2}}\exp\left(-\frac{R^{2}}{4Dt}\right)+T_{0}$$

Then, the heat produced by the studied point *A*_0_ at any time *t* due to the point source at the moment τ can be expressed as follows:

(3) $$dT(x,y,z,t)=\frac{Pd\tau }{4\rho c{\left[\pi D(t-\tau )\right]}^{3/2}}\exp\left(-\frac{R^{2}}{4D(t-\tau )}\right)$$

where *R* represents the distance between the point source and the object being studied, $R^{2}={\left[x+V(t-\tau )\right]}^{2}+y^{2}+z^{2}$.

Integrating the temperature of the moving point heat source at each moment over the entire time domain, the temperature response of a continuous moving point heat source on the surface of a semi-infinite medium in its interior can be obtained [27,28]:

(4) $$T_{P}(x,y,z,t)=\int_{0}^{t}\frac{P}{4\rho c{\left[\pi D(t-\tau )\right]}^{3/2}}\exp\left(-\frac{{\left[x+v(t-\tau )\right]}^{2}+y^{2}+z^{2}}{4D(t-\tau )}\right)d\tau +T_{0}$$

Equation (4) gives the mathematical representation for the temporary temperature distribution created by the moving point heat source on the surface of a semi-infinite medium, which gives the change process of the thermal environment within the linearly moving point heat source from its generation to the time *t*.

The diameter of the laser spot in the LPBF process is typically smaller than 100 μm, and the energy of the spot is generally regarded as Gaussian distribution. Using Green’s function method, the solution for the heat source on a Gaussian surface is determined instantaneously; the analytical solution of the temperature field of linearly moving Gaussian surface heat source can be described as follows [29]:

(5) $$T_{S}(x,y,z,t)=\frac{\alpha P}{\pi \rho c\sqrt{4\pi \kappa }}\int_{0}^{t}\frac{{\left(t-\tau \right)}^{-1/2}}{2D(t-\tau )+\sigma_{1}^{2}}\exp\left(-\frac{(x-v\tau )+y^{2}}{4D(t-\tau )+2\sigma_{l}^{2}}-\frac{z^{2}}{4D(t-\tau )}\right)d\tau +T_{0}$$

where the standard deviation of the Gaussian distribution of the laser spot is denoted by the symbol $\sigma_{l}$.

For a heat source distributed throughout a volume with thermal intensity of $Q_{V}\left(x^{\prime },y^{\prime },z^{\prime }\right)=\frac{2^{5/2}\alpha P}{\pi^{3/2}r^{3}}\exp\left(-\frac{2\left(x^{2}+y^{2}+z^{2}\right)}{r^{2}}\right)$, and the hemisphere radius of *r*, the formula for calculating the laser beam heat source for a Gaussian distribution over the entire volume can be further simplified as follows [30]:

(6) $$T_{V}(x,y,z,t)=\int_{0}^{t}\frac{2^{5/2}\alpha P}{\pi^{3/2}\rho c{\left(r^{2}+8D\left(t-\tau \right)\right)}^{3/2}}\times \exp\left(-\left(\frac{2{\left(x-v_{x}\tau \right)}^{2}}{r^{2}+8D\left(t-\tau \right)}+\frac{2{\left(y-v_{y}\tau \right)}^{2}}{r^{2}+8D\left(t-\tau \right)}+\frac{2z^{2}}{r^{2}+8D\left(t-\tau \right)}\right)\right)d\tau +T_{0}$$

#### 2.2.2. Boundary Condition Setting

During the LPBF process, complicated heat transfer occurs, such as heat convection, heat radiation, and heat conduction. Hence, it is necessary to establish suitable boundary conditions to obtain the accurate temperature distribution.

First, the initial state of the LPBF process is represented as follows:

(7) $${T(x,y,z,t)|}_{t=0}=T_{0}$$

where $T_{0}$ is the initial temperature, which is taken as 20 °C in this paper.

Secondly, the temperature distributions at the part boundaries as well as the non-uniform convective and radiative heat losses need to be considered. In the process illustrated in Figure 1, the metal undergoes melting when the laser is directed towards the metal powder, resulting in the formation of a molten pool on the surface. This process involves the transfer of heat by convection and radiation. Therefore, the boundary conditions at the upper surface of the molten pool can be defined as follows:

(8) $$-k\frac{\partial T}{\partial z}=h\left(T-T_{0}\right)+\sigma \varepsilon \left(T^{4}-T_{0}^{4}\right)$$

where *h* is the convection coefficient, which is related to the properties of the fluid, flow velocity, and the heat exchange area between the fluid and the air at the surface of the molten pool. Herein, it is taken as 10 W m^−2^ K^−1^. *σ* is the Boltzmann constant, which is taken as 5.6697 × 10^−8^ W m^−2^ K^−4^. *ε* is the radiation coefficient, which is related to the surface characteristics and the temperature of the molten pool. This is taken as 0.9 in this paper [31]. There is no relationship between the convection coefficient *h* and the radiation coefficient *ε*.

In this study, factors such as material evaporation, recoil pressure, surface tension, and heat dissipation due to the evaporation effect are not considered [31]. The semi-infinite medium configuration is a basic assumption in this study. At the same time, an assumption is made in the study that the thermophysical parameters of the powder and those of the substrate entities are consistent and do not change with temperature. The heat dissipation resulting from thermal convection and radiation is deemed to take place at the upper surface of the powder bed as a consequence of the interaction between the laser and the powder. Nevertheless, according to Refs. [32,33], the convective heat loss on the top surface is quite minor during the LPBF of metals compared to thermal conduction and radiation. This is because it has a little impact on the thermal behavior due to the high temperature gradients. In this study, the powder is considered a homogeneous solid, and its porosity is not taken into account. This assumption was made in order to reduce the complexity of the model and to focus on the sensitivity analysis of the temperature field. Nevertheless, the porosity of the powder may significantly influence the temperature distribution and the final material properties during the laser powder bed melting process. Consequently, this assumption represents a significant limitation of this study.

#### 2.2.3. Thermophysical Parameters of Materials

In the realm of analytical calculations, addressing the alterations in thermophysical parameters for metal powder undergoing solid transformation presents increased complexity. The composites of the Ti6Al4V alloy material are listed in Table 1. The analytical method relies on the assumption that material physical property parameters remain constant. In light of this, this paper adopts the material properties as described in Ref. [19], which are calibrated by the experimental data. A comprehensive overview of Ti6Al4V and corresponding material properties are listed in Table 2.

## 3. Results and Discussion

### 3.1. Comparison of Heat Source Models

#### 3.1.1. Experimental Condition

**Figure 4:**
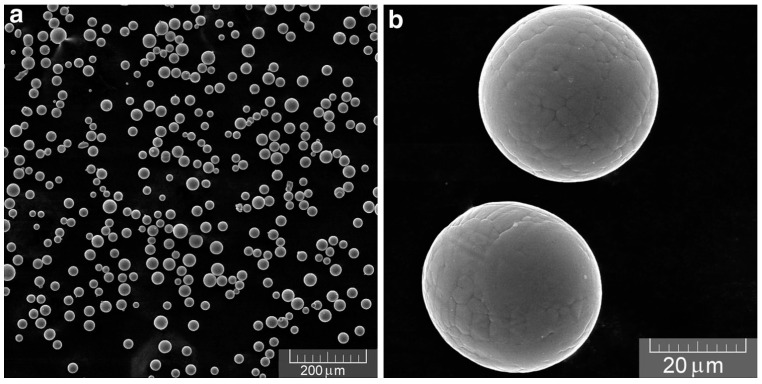
(**a**) Low-magnification SEM micrograph of Ti6Al4V powder. (**b**) High-magnification SEM micrograph of powder particles [34].

First, the above heat source models are verified by comparing the experimental data in Ref. [34]. The Ti6Al4V metallic material was adopted. The process parameters are summarized in Table 3. The morphology of the Ti6Al4V metal powder was observed using Scanning Electron Microscopy (SEM), which exhibited the particle size distribution as 15–45 μm, as shown in Figure 4. For the scanning process, a fiber laser model EOS M270 was selected, with a maximum power output of 200 W and a spot diameter not less than 100 μm. In this test, the laser beam’s focal point had a radius of 50 μm, and the powder layer’s thickness was set at 30 μm.

Under the condition of a 3.3 mm thickness of the Ti6Al4V substrate, a single-pass, single-layer printing was conducted according to the specified processing parameters, with a scanning trajectory length of 100 mm. To assess and compare the shapes of molten pools generated by different heat sources under various processing parameters, the processing parameters were uniformly converted to energy density *E* (J/mm^3^) with the same magnitude as shown in Equation (9); the specific values can be seen in Table 3.

(9) $$E=\frac{P}{2RV\delta }$$

where δ is the thickness of powder layer; *R* is the laser spot radius.

**Figure 5:**
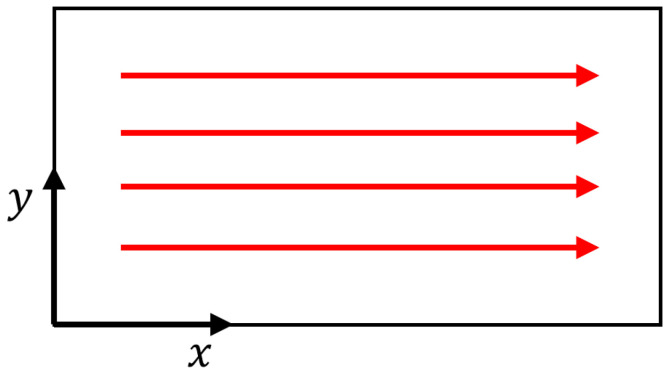
Schematic diagram illustrating the scanning trajectory.

In the LPBF process, the initial phase involves the interaction of laser light with the surface of the metal powder. The metal powder absorbs and reflects a significant portion of the incident light. The resulting absorbed heat induces the melting of the metal powder, leading to the release of gases from voids between the particles [35]. Subsequently, the particles undergo melting and condensation, culminating in the formation of a solid material. This transformation signifies the shift of the metal from a powdered state to a solid form. Figure 5 provides a schematic representation of the scanning trajectory.

#### 3.1.2. Results and Discussion of Molten Pool Size

**Figure 6:**
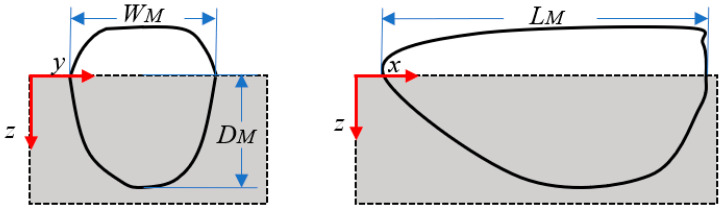
The schematic of the molten pool shape and dimension.

This study presents the relevant dimensions of the molten pool, including the length *L_M_*, the width *W_M_*, and the depth *D_M_*, as shown in Figure 6. This paper uses the width, the depth, and the length to represent the size of the molten pool. The subsequent analysis will explore the applicable heat source modes under different laser energy density conditions through contrasting the simulated predictions of the dimensions of the molten pool with the experimental measurements [36].

### 3.2. Sensitivity Analysis of Temperature for Gaussian Surface Heat Source

#### 3.2.1. Effects of Process Parameters

**Figure 7:**
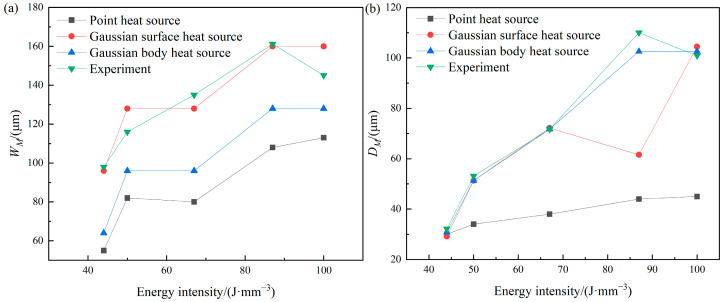
The comparison of the molten pool dimensions: (**a**) the molten pool width; (**b**) the molten pool depth.

Figure 7 illustrates the comparison of the dimensions of the molten pool between the predicted results and the experimental data. The size of the build part and the measurement location can be seen in Ref. [37]. Regarding the molten pool width depicted in Figure 7a, it shows that the predicted trends of the three heat source models are consistent with the experimental values, compared to the point heat source, the Gaussian surface heat source and the hemispherical Gaussian body heat source show better agreement with the experimental values. The maximum prediction errors of the Gaussian body and surface heat source are 34.64% and 11.78%, respectively, while the minimum prediction errors are 10.37% and 1.12%, and the average prediction errors are 20.60% and 5.70%. The preceding comparison indicates that the prediction accuracy of the Gaussian surface heat source is superior to that of the Gaussian body heat source. In the LPBF process, the laser primarily exhibits the properties of a Gaussian surface heat source, focusing the energy on the top surface of the molten pool. Conversely, the body heat source converts all input energy into an internal heat source with a distinct shape, resulting in the energy being distributed along the molten pool’s depth. However, the energy present on the molten pool’s surface is relatively limited compared to that of a Gaussian surface heat source. Consequently, the Gaussian surface heat source is superior to the body heat source in predicting the molten pool’s width.

Regarding the depth of the molten pool depicted in Figure 7b, it shows that the prediction accuracy of the point heat source is obviously inferior to those of the other two heat sources. From the comparison, it is obvious that, when the laser energy density is between 40 and 70 J/mm^3^, the prediction accuracy of the Gaussian surface and body heat source is similar, and the average prediction errors are 4.427% and 2.613%, respectively. When the laser energy density ranges from 70 to 90 J/mm^3^, the Gaussian body heat source demonstrates better prediction accuracy compared to the Gaussian surface heat source, with average prediction errors of 44.05% and 6.82%, respectively. This occurs because, as the energy density rises, the energy penetration in the depth direction of the molten pool is greater for the Gaussian body heat source compared to the surface heat source.

**Figure 8:**
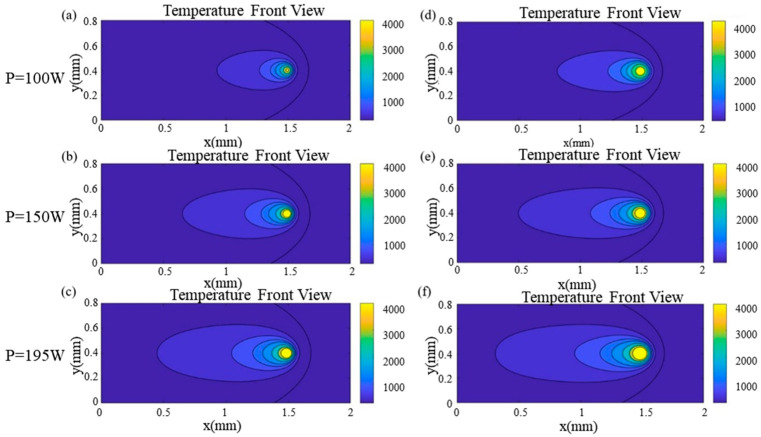
Temperature distribution under different laser powers (V = 750 mm/s, t = 0.85 ms): (**a**–**c**) Gaussian surface heat source; (**d**–**f**) Gaussian body heat source.

Figure 8 shows the temperature distributions of the Gaussian surface and body heat source under three laser power conditions (100 W, 150 W, and 195 W), using a scanning duration of 0.85 ms and a scanning velocity of 750 mm/s. Figure 8a–c represent the Gaussian surface heat source mode, while Figure 8d–f represent the Gaussian body heat source mode. As the laser power increases, the maximum temperature for both the Gaussian surface heat source and the Gaussian body heat source also rises. However, the maximum temperature with the Gaussian surface heat source surpasses that with the Gaussian body heat source. Due to the penetration of some energy from the Gaussian body heat source into the build part’s depth, the maximum temperature of the Gaussian body heat source is consequently lower compared to that of the surface heat source.

**Figure 9:**
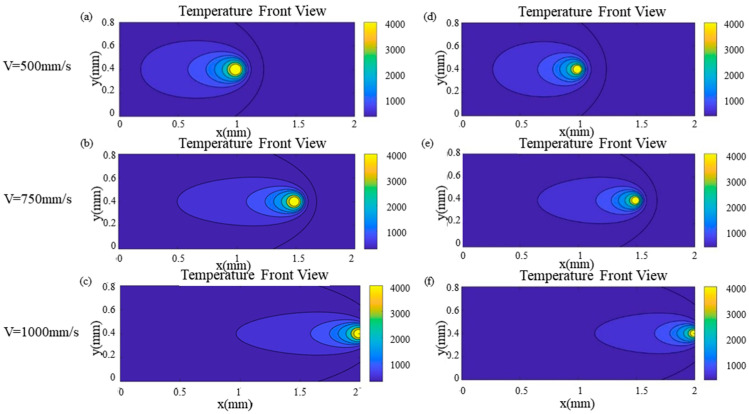
Temperature distribution under different scan speeds (P = 150 W, t = 0.002 ms): (**a**–**c**) Gaussian surface heat source; (**d**–**f**) Gaussian body heat source.

Figure 9 shows the temperature distributions of the Gaussian surface and body heat source under different scanning speeds (500 mm/s, 750 mm/s, and 1000 mm/s), using a scan time of 0.002 ms and a laser power of 150 w. Figure 9a–c on the left represent the Gaussian surface heat source, while Figure 9d–f on the right represent the Gaussian body heat source. The temperature profiles in Figure 9 reveal that, as the scanning speed rises, the maximum temperatures for both the Gaussian surface and body heat source decline. Similarly to Figure 8, the maximum temperature with the Gaussian surface heat application exceeds that of the Gaussian body heat application.

In order to quantitatively assess the factors affecting temperature, the suitable process parameters provided in Ref. [34] are adopted, using a laser power of P = 100 W and a scanning velocity of V = 750 mm/s, and the material thermophysical property parameters as shown in Table 2. This set of parameters is chosen as the standard set of parameters and the predicted results of the other parameter sets were compared with the standard set of results.

**Figure 10:**
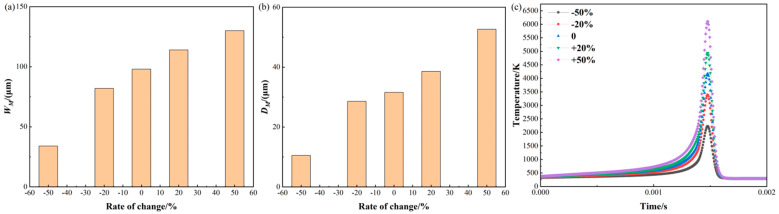
Molten pool size and temperature variation with laser power: (**a**) molten pool width; (**b**) molten pool depth; (**c**) temperature history.

Figure 10 shows the changes in molten pool size and temperature in response to varying laser power. The molten pool’s dimensions are 98.16 μm in width and 31.58 μm in depth, following the standard value. For the width as shown in Figure 10a, decreasing the laser power by 50% and 20% leads to a reduction in the width by 64.99% and 16.01%, correspondingly. And the widths are 34.36 μm and 82.44 μm. However, increasing the laser power by 50% and 20% results in a corresponding expansion of the width by 32.65% and 16.32%, correspondingly. And the widths are 130.21 μm and 114.18 μm, respectively. From the above predicted data, it is evident that, when the laser power reduced substantially, the width changed significantly compared to when the laser power increased. This occurs when the laser power is greatly reduced, the powder material cannot be completely melted, and the width significantly decreases. Although the laser power is significantly increased, the occurrence of evaporative convection in the molten pool does not impact its width. However, it can lead to the formation of defects inside the molten pool.

For the molten pool depth as shown in Figure 10b, a reduction in laser power of 50% and 20% results in a corresponding reduction in depth of 66.67% and 9.50%, respectively. And the depths are 10.53 μm and 28.58 μm. However, an increase in laser power of 50% and 20% results in a 66.67% and 22.17% increase in depth. And the depths are 38.58 μm and 52.63 μm, respectively. The laser power has a direct impact on the amount of heat that is transferred to the molten pool. As a result, it influences both the size and the temperature distribution of the pool. By increasing the laser power, the molten pool is subjected to more intense heating, leading to an expansion in both its depth and width.

Regarding the temperature history depicted in Figure 10c, at the standard value, the maximum temperature is 4168.41 K; when the laser power is reduced by 50% and 20%, the maximum temperature is reduced by 46.49% and 18.47%. And the maximum temperatures are 2230.71 K and 3393.33 K, respectively. However, when the laser power is increased by 50% and 20%, the maximum temperature is increased by 46.49% and 18.69%, respectively. Therefore, it is found that increasing the laser power results in the metal powder absorbing more energy, leading to higher temperatures.

**Figure 11:**
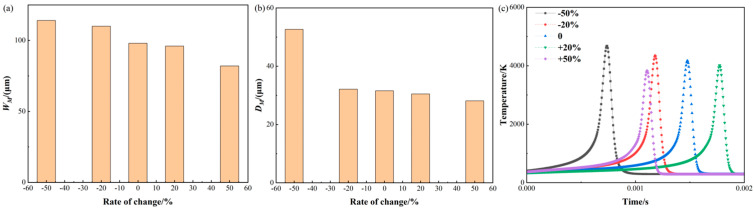
Molten pool size and temperature variation with scan speed: (**a**) molten pool width; (**b**) molten pool depth; (**c**) temperature history.

Figure 11 shows changes in molten pool size and temperature in response to varying the scan speed. Regarding the molten pool width depicted in Figure 11a, decreasing the scan speed by 50% and 20% leads to a reduction in the width by 16.13% and 12.05%, correspondingly. And the widths are 113.99 μm and 109.99 μm, respectively. However, increasing the scan speed by 50% and 20% results in a corresponding expansion of the molten pool width by 16.45% and 2.04%, correspondingly. And the widths are 82.01 μm and 96.16 μm.

For the molten pool depth as shown in Figure 11b, a reduction in scan speed of 50% and 20%, respectively, results in an increase in depth of 66.67% and 1.64%. And the depths are 52.63 μm and 32.10 μm, respectively. However, an increase in scan speed of 50% and 20% results in a 11.02% and 3.42% increase in depth. And the depths are 28.1 μm and 30.50 μm, respectively. The reason for these changes can be attributed to the fact that the scan speed affects the laser action time on the powder and the solidification rate of the molten pool, which in turn affects the size and temperature distribution of the molten pool. As the scan speed decreases, the laser action time on the powder increases and the molten pool spreads more easily, leading to an increase in width and depth, while the opposite is true as the scan speed increases.

For the temperature history as shown in Figure 11c, the time value is taken as 0.001 s for a scan speed of +50%. When the scan speed is reduced by 50% and 20%, the maximum temperature is increased by 12.26% and 4.38%. And the maximum temperatures are 4679.57 K and 4351.02 K, respectively. However, when the scan speed is increased by 50% and 20%, the maximum temperature is reduced by 8.10% and 3.64%, respectively.

From the discussion above, it is found that the width is more sensitive to scan speed than its depth. The scan speed is related to the time of localized area heating. Increasing the scan speed can reduce the print time and cost. However, it also increases the likelihood of print defects.

**Figure 12:**
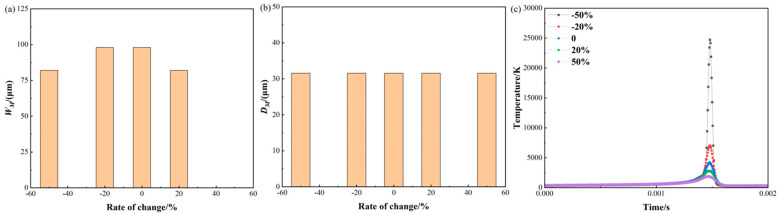
Molten pool size and temperature variation with laser spot radius: (**a**) molten pool width; (**b**) molten pool depth; (**c**) temperature history.

Figure 12 shows the molten pool size and temperature variation with the laser spot radius. For the molten pool width as shown in Figure 12a, when the laser spot radius is reduced by 50% and 20%, the width of the molten pool is reduced by 16.47% and 0.16%. And the widths of the molten pool are 81.99 μm and 98.00 μm, respectively. However, when the laser spot radius is increased by 20%, the width of the molten pool is reduced by 16.44%. And the width of the molten pool is 82.02 μm.

For the molten pool depth as shown in Figure 12b, the depth of the molten pool remains constant throughout the process. This could be attributed to the change in heat distribution caused by the alteration of the laser spot radius. A larger laser spot may distribute heat more evenly across the surface, while a smaller spot may concentrate heat in a smaller area below the surface.

For the temperature history as shown in Figure 12c, when the laser spot radius is reduced by 50%, the maximum temperature is 24,723.31 K. The heat source model exhibits an over-concentration problem at this laser spot radius, resulting in very high temperatures in a short period of time. This may be attributed to the non-uniform distribution of heat in the model or an excessively fast rate of heat conduction [38]. However, when the laser spot radius is increased by 50% and 20%, the maximum temperature is reduced by 54.88% and 18.55%, respectively. Additionally, the absorption rate of the laser may vary with the spot size, which affects the formation of the molten pool and leads to higher temperature variations. The spot size of the laser can be modified by varying the manufacturing focal offset without changing any other processing parameters.

#### 3.2.2. Effects of the Material Properties

**Figure 13:**
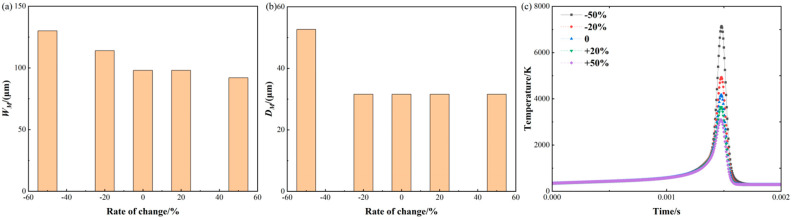
Molten pool size and temperature variation with density of the material: (**a**) molten pool width; (**b**) molten pool depth; (**c**) temperature history.

Figure 13 shows the molten pool size and temperature variation with the density of the material. For the molten pool width as shown in Figure 13a, when the density of the material is reduced by 50% and 20%, the width of the molten pool is increased by 32.65% and 16.33%. And the widths of the molten pool are 130.21 μm and 114.19 μm, respectively. However, when the density of the material is increased by 50% and 20%, the width of the molten pool is reduced by 16.60% and 0.16%. And the widths of the molten pool are 81.87 μm and 98.00 μm, respectively.

For the molten pool depth as shown in Figure 13b, when the density of the Ti6Al4V powder is reduced by 50% and 20%, the depth of the molten pool is increased by 66.67% and 1.64%. And the depths of the molten pool are 52.63 μm and 32.10 μm, respectively. However, when the density of the material is increased by 50% and 20%, the depths of the molten pool are 32.10 μm and 32.10 μm, respectively. The reason for these changes can be attributed to the fact that the density of the material affects the build-up of the powder particles and the effect of the laser on the powder, which in turn affects the size and temperature distribution of the molten pool. As the density of the material decreases, there is less accumulation of powder particles, the laser effect is more pronounced, and the molten pool becomes more diffuse, leading to an increase in width and depth.

For the temperature history, as shown in Figure 13c, when the density of the Ti6Al4V metal powder is reduced by 50% and 20%, the maximum temperature is increased by 71.42% and 18.32%. And the maximum temperatures are 7145.4112 K and 4932.153 K, respectively. However, when the density of the material is increased by 50% and 20%, the maximum temperature is reduced by 25.61% and 12.52%, respectively. The density of Ti6Al4V powder can be influence by the powder particle size and the packing style.

**Figure 14:**
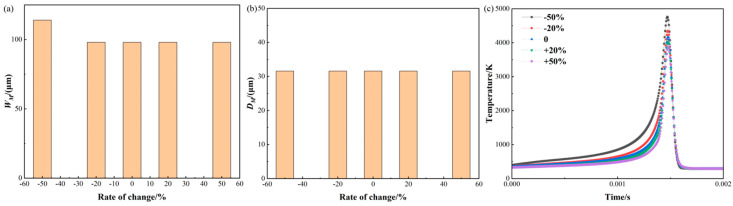
Molten pool size and temperature variation with thermal conductivity: (**a**) molten pool width; (**b**) molten pool depth; (**c**) temperature history.

Figure 14 shows the molten pool size and temperature variation with thermal conductivity. For the molten pool width as shown in Figure 14a, when the thermal conductivity is reduced by 50% and 20%, the width of the molten pool is increased by 16.13% and 0.16%. And the widths of the molten pool are 113.99 μm and 98.00 μm, respectively. However, when the thermal conductivity is increased by 50% and 20%, the width of the molten pool remains the same.

For the molten pool depths as shown in Figure 14b, the depth at −50%, −20%, +20%, and +50% does not change significantly; this phenomenon is likely attributable to the enhanced thermal conductivity of the material, which facilitates the rapid transfer of heat within it. But, actually, there is no significant variation and the width remains constant. The limited impact of thermal conductivity on the depth of the molten pool may be attributed to either its less significant influence compared to other components, or the counteractive effect of other parameters and material qualities within this range.

Regarding the temperature history depicted in Figure 14c, when the thermal conductivity is reduced by 50% and 20%, the maximum temperature is increased by 14.27% and 4.38%. And the maximum temperatures are 4763.22 K and 4351.138 K, respectively. However, when the thermal conductivity is increased by 50% and 20%, the maximum temperature is reduced by 6.79% and 3.27%, respectively. Different powder morphologies can affect the thermal conductivity by changing the level of connection between the particles and the gap.

**Figure 15:**
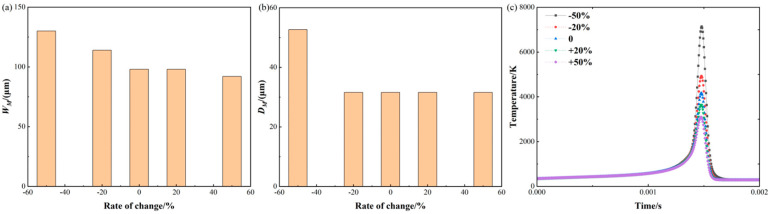
Molten pool size and temperature variation with specific heat: (**a**) molten pool width; (**b**) molten pool depth; (**c**) temperature history.

Figure 15 shows the molten pool size and temperature variation with specific heat. For the molten pool width as shown in Figure 15a, a reduction in specific heat of 50% and 20%, respectively, results in an expansion of the width by 32.64% and 16.33%. And the widths are 130.20 μm and 114.19 μm, respectively. However, an increase in specific heat of 50% and 20% results in a 16.33% and 0.16% reduction in width. And the widths are 82.13 μm and 98.00 μm, respectively.

For the molten pool depth as shown in Figure 15b, when the specific heat is reduced by 50% and 20%, the depth is increased by 66.67% and 0%. And the depths are 52.63 μm and 31.58 μm, respectively. However, an increase of 50% and 20% in specific heat does not result in a significant change in depth. A reduction in specific heat indicates that the material requires less energy to raise its temperature. For a material such as Ti6Al4V, this may result in the melt pool boundary reaching the melting temperature more easily, resulting in a larger melt pool. Conversely, an increase in specific heat indicates that the material requires more energy to raise its temperature. This may result in the melt pool boundaries having more difficulty in reaching the melting temperature, thus forming a smaller melt pool. This suggests that the change in specific heat does not have a consistent effect on the depth of the molten pool and may be influenced by other factors. Even if the specific heat of the material changes, the effect on the depth of the melt pool may be relatively minor, resulting in insignificant alterations.

For the temperature history as shown in Figure 15c, when the specific heat is reduced by 50% and 20%, the maximum temperature is increased by 71.42% and 18.32%. And the maximum temperatures are 7145.41 K and 4932.15 K, respectively. However, when the specific heat is increased by 50% and 20%, the maximum temperature is reduced by 25.61% and 12.52%, respectively.

#### 3.2.3. Combination Effects

The properties of the build part can be significantly affected by laser-related properties, such as laser spot size, laser power, and scan parameters including scan strategy and scan speed. These parameters, in turn, can be used to determine the energy density or energy input. The performance and quality of components fabricated through LPBF are also significantly affected by these parameters. The thermophysical properties of a material provide information on its behavior under thermal application. In the LPBF process, these properties affect laser–metal interactions, including melting and heat transfer [37,39]. The most influential thermophysical properties in the LPBF process are density, specific heat, thermal conductivity, and the thermal diffusion coefficient. These properties are related to either thermodynamics or heat transfer phenomena. For instance, specific heat is a thermodynamic property, whereas thermal conductivity is a property that influences heat transfer.

Under the quantitative description of different process parameters (laser power, scan speed, laser spot radius) and material properties (density, thermal conductivity, specific heat) on the molten pool size and peak temperature derived above, the sensitivity analysis of the above parameters on the width, depth, and maximum temperature is carried out by using mathematical analysis and SPSS software 27.0 to derive the influence of each parameter on the width, depth, and maximum temperature weights, and draw radar diagrams.

**Figure 16:**
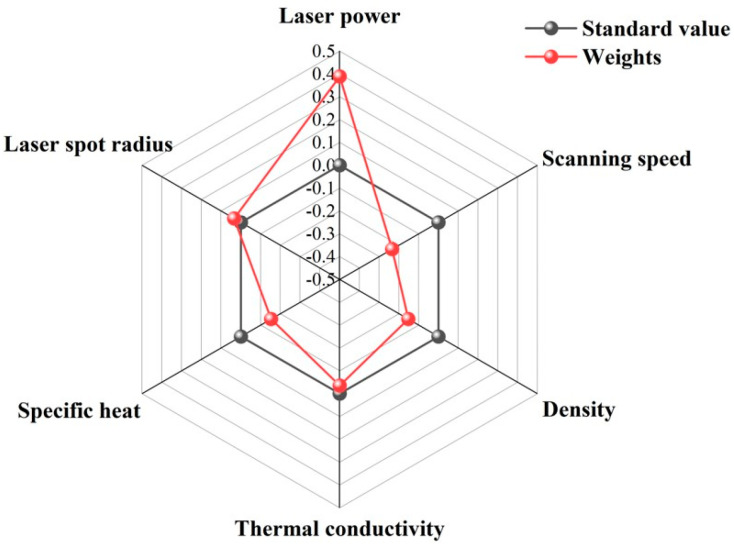
Weighting effects of varying process parameters and material properties in molten pool width.

**Figure 17:**
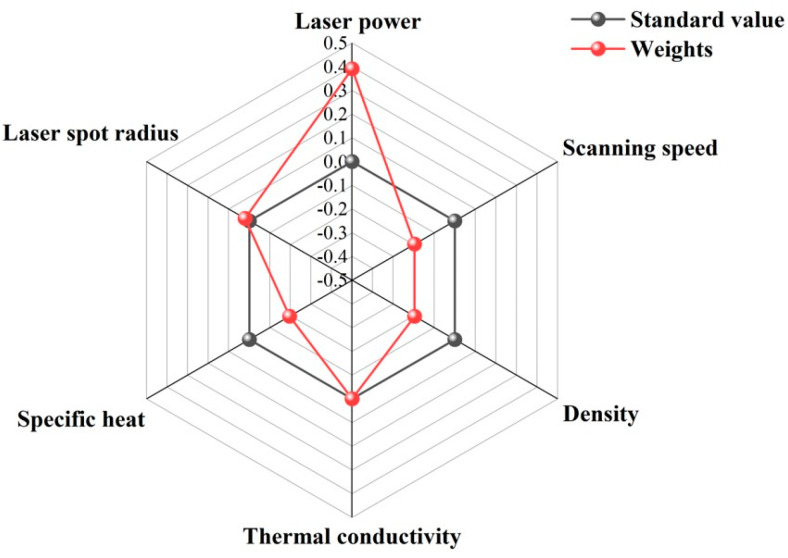
Weighting effects of varying process parameters and material properties in molten pool depth.

**Figure 18:**
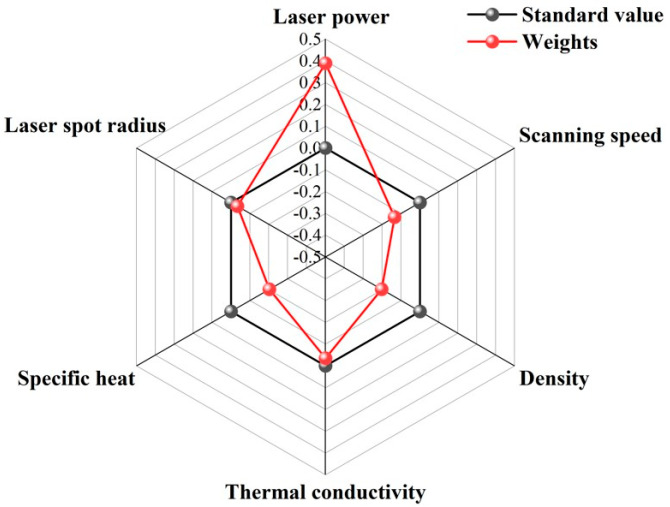
Weighting effects of varying process parameters and material properties in peak temperature.

From Figure 16, Figure 17 and Figure 18, it is clearly seen that the influence of each parameter on the width, depth, and maximum temperature weights in the standard value of the part of the circle, which indicating that the parameter has a positive or negative effect. For example, the laser power outside the standard value of the circle indicates that it has a positive effect. With the increase of the laser power, the width, depth, and maximum temperature will also increases. For the width, it can be seen that the laser power, scan speed, density, thermal diffusivity, specific heat, and laser spot radius sensitivity weights are 0.389, 0.235, 0.153, 0.035, 0.153, and 0.034, in order; for the depth, it can be seen that the laser power, scan speed, density, thermal diffusivity, specific heat, and laser spot radius sensitivity weights are 0.391, 0.196, 0.196, 0.005, 0.191, and 0. 021; for the maximum temperature, it can be seen that the laser power, scan speed, density, thermal diffusivity, specific heat, and laser spot radius sensitivity weights are 0.389, 0.215, 0.163, 0.035, 0.163, and 0.034, in turn. According to the weight analysis results, the laser power, scan speed, density, thermal diffusivity, specific heat, and laser spot radius are the key parameters affecting the width, depth, and maximum temperature of the molten pool in the LPBF process. Among them, laser power has the most significant effect on these parameters, followed by scan speed and density, while thermal diffusivity, specific heat, and laser spot radius have relatively small effects. These conclusions provide important theoretical and practical guidance for the optimization and control of LPBF process parameters.

## 4. Conclusions

Based on the validated temperature analytical predictive model, the energy density range applicable to different heat sources was investigated and verified by experimental comparison. The influence weights of process parameters and material parameters on molten pool size and temperature history were investigated in detail; the conclusions are summarized as following:

(1) Under the same processing parameters, the prediction accuracy of the width is higher for the Gaussian surface and body heat source models compared to the point heat source. The average prediction error of the surface heat source model is 5.696%, while the average prediction error of the body heat source model is 20.598% over the tested range of laser power between 100 W and 195 W, and scan speed between 500 mm/s and 1200 mm/s.

In addition, the prediction accuracies of the depth in different energy density ranges also show differences; when the laser energy density is between 40 and 70 J/mm^3^, the prediction accuracy of the Gaussian surface and body heat source is similar, and the average prediction errors are 4.427% and 2.613%, respectively. When the laser energy density is between 70 and 90 J/mm^3^, the prediction accuracy of the Gaussian body heat source is superior to that of Gaussian surface heat source.

(2) The effects of laser power, scan speed, material density, and material specific heat on the molten pool width, depth, and maximum temperature are more significant than the laser spot radius and the material thermal conductivity, with laser power having the highest percentage of influence, while the effects of thermal conductivity and laser spot radius are relatively small. This provides an important reference for the optimization and control of parameters in the LPBF process.

(3) The weighting effects of varying process parameters and material properties in the molten pool size and temperature are quantitatively described, which will provide a theoretical basis for the process optimization of LPBF.

## Figures and Tables

**Table 1:** Ti6Al4V alloy powder material elemental mass fraction (wt.%).

C	O	Ni	H	Fe	Al	V	Ti	Other
0.02	0.11	0.02	0.0034	0.19	6.5	3.9	surplus	<0.1

**Table 2:** Material properties of Ti6Al4V [19,34].

Material Properties	Ti6Al4V
Liquidus temperature *T_L_* (K)	1923
Solidus temperature *T_S_* (K)	1877
Thermal conductivity *k* (W m^−2^ K^−1^)	31.4
Specific heat *c* (J kg^−1^ K^−1^)	830
Density ρ (kg m^−3^)	4200

**Table 3:** Parameters of single-pass scanning process of LPBF.

Laser Power *P* (W)	Scan Speed *V* (mm s^−1^)	Powder Layer Thickness δ (μm)	Energy Density *E* (J mm^−3^)
100	500,750	30	67,44
150	500,750,1000,1200	30	100,67,50,42
195	500,750,1000,1200	30	130,87,65,54

## Data Availability

All the data in the tests of this study have been listed in the paper.
